# Conceptions of learning factors in postgraduate health sciences master students: a comparative study with non-health science students and between genders

**DOI:** 10.1186/s12909-018-1227-x

**Published:** 2018-06-07

**Authors:** Fernando Campos, Miguel Sola, Antonio Santisteban-Espejo, Ariane Ruyffelaert, Antonio Campos-Sánchez, Ingrid Garzón, Víctor Carriel, Juan de Dios Luna-Del-Castillo, Miguel Ángel Martin-Piedra, Miguel Alaminos

**Affiliations:** 1 Group of Tissue Engineering, Department of Histology (Tissue Engineering Group), University of Granada and research institute ibs.Granada, Granada, Spain; 2 Ph.D. student of Biomedicine Program, University of Granada, Granada, Spain; 3 Andalusian Health Service, Puerta del Mar Hospital, Cádiz, Spain; 4 Department French Philology, University of Granada, Granada, Spain; 5 Group of Research in Education HUM-672, University Granada, Granada, Spain; 6 Biostatistics, School of Medicine, University of Granada, 18071 Granada, Spain; 7 Department of Histology, School of Medicine, Avda de la Ilustración, 11, 18016 Granada, Spain

**Keywords:** Conceptions of learning, Postgraduate master students, Health sciences

## Abstract

**Background:**

The students’ conceptions of learning in postgraduate health science master studies are poorly understood. The aim of this study was to compare the factors influencing conceptions of learning in health sciences and non-health sciences students enrolled in postgraduate master programs in order to obtain information that may be useful for students and for future postgraduate programs.

**Methods:**

A modified version of the Learning Inventory Conception Questionnaire (COLI) was used to compare students’ conception learning factors in 131 students at the beginning of their postgraduate studies in health sciences, experimental sciences, arts and humanities and social sciences.

**Results:**

The present study demonstrates that a set of factors may influence conception of learning of health sciences postgraduate students, with learning as gaining information, remembering, using, and understanding information, awareness of duty and social commitment being the most relevant. For these students, learning as a personal change, a process not bound by time or place or even as acquisition of professional competences, are less relevant. According to our results, this profile is not affected by gender differences.

**Conclusions:**

Our results show that the overall conceptions of learning differ among students of health sciences and non-health sciences (experimental sciences, arts and humanities and social sciences) master postgraduate programs. These finding are potentially useful to foster the learning process of HS students, because if they are metacognitively aware of their own conception or learning, they will be much better equipped to self-regulate their learning behavior in a postgraduate master program in health sciences.

**Electronic supplementary material:**

The online version of this article (10.1186/s12909-018-1227-x) contains supplementary material, which is available to authorized users.

## Background

Learning has been defined as a multidimensional construct including references to learning processes, learning benefits, learning demands, learning goals, etc. [1]. According with Säljö conceptions of learning specifically refer to the beliefs and understanding that learners have about learning [2]. Since then, two main conceptions of learning have been basically categorized: a surface conception involving the acquisition and reproduction of knowledge (learning is acquisition of facts) and a deep conception of learning that involves abstraction of meaning and personal change (learning is understanding and seeing things in new way) [3–10]. Research in this area has indicated that conceptions of learning influences not only students’ motivation to learn but also the cognitive strategies they adopt in their learning process and their academic achievement [11–13]. Six main factors have been identified and considered as the best model to interpret conceptions of learning for students coming from several cultural groups [8]: (a) gaining information; (b) remembering, using, and understanding information; (c) a duty; (d) personal change; (e) a process not bound by time or place and (f) social competence.

According to their different conceptions of learning, students would design their own strategy in relation to the objectives and teaching skills they should achieve [8, 13]. These achievements can be predicted based on the type of strategy they have previously designed [11, 14, 15]. Recently, several studies have demonstrated that teachers’ conceptions of learning also affect, although indirectly, students’ learning outcomes [16–18].

Research in students’ conceptions of learning has been mainly carried out in primary and secondary education [1, 19] and in undergraduate students [4, 5]. However, few studies related to conception learning have been carried out on postgraduate students [20–23]. Studies related to conceptions of learning usually made use of the Conceptions of Learning Inventory (COLI), which is designed to analyze the six factors described by Purdie and Hattie [8]. However, none of these studies focused on the analysis of components related to learning as acquisition of professional competences, which is especially important in postgraduate students, since these competences are in their near horizon. For this reason, we have elaborated and added to the inventory a 7th factor related to professional competences. The modified questionnaire demonstrated reliability and validity as described below. Furthermore, as pointed out by Haggis [24], there is very little research on the relationship between theoretical ideas about adult learning and the learning process described by the adult students themselves, particularly in the context of higher education. Consequently, the design of postgraduate educational programs is not always related to learning strategies that arise from the different conceptions of learning. This is especially important in medical and health sciences education in which postgraduate studies are very well regulated. In addition, self-directed learning is a key element in postgraduate studies not only to acquire new knowledge but also as a way of acquiring a competence-based education [25–28].

In a pilot study, we have demonstrated the existence of some significant differences in the conceptions of learning between students of health sciences and students of social sciences who begin their postgraduate studies [29]. A better knowledge on the conceptions of learning of health sciences postgraduate students and the incidence of factors influencing those conceptions of learning would allow a more appropriate design of postgraduate programs in order to acquire knowledge and skills related to these studies.

In the present work, we performed a comparative study between students of a health sciences master and students enrolled in other postgraduate master programs in experimental sciences, arts and humanities and social sciences to determine the profiles of similarity and discrepancy in their conceptions of learning in order to obtain a valuable information contributing to a better design of future postgraduate programs. Previous studies showed that culture and discipline might influence the students’ conceptions of learning, although results are controversial [20, 30]. In addition, research about the influence of gender in several aspects of medical education is increasing [31, 32], but its influence on the conceptions of learning has been poorly studied [8, 33]. For this reason, the incidence of gender of the different components of the conceptions of learning in the postgraduate students was also investigated in the present study. Since factors related to conceptions of learning may influence the learning process and therefore, the way we should teach, research on specific features of conceptions of learning in health science students as compared to non-health science students could be useful in biomedical education.

## Methods

### Design of the study

The present investigation was carried out to compare the conceptions of learning of students at the beginning of their postgraduate studies. To compare the results among different groups of postgraduate students, the study was performed during the same course and in the same period of the year. All the students received the same information about the goals of the study and the procedure to be used.

### Sample

The study was done at the University of Granada (Spain). The sample consisted in 131 (73 males and 58 females) postgraduate students (average 27.3 ± 5.4 years of age) enrolled in four full-time master’s degrees in health sciences (HS), experimental sciences (ES), arts and humanities (AH) and social sciences (SS). Students accessing each type of master degree have previously received their graduation in curricula related to each master area. Thirty-two students (21males and 11 females) were enrolled in the master studies in HS; 35 (19 males and 16 females) in ES; 34 (20 males and 14 females) in AH and 30 (13 males and 17 females) in SS. Participation of the students was voluntary and consistent with the procedures of the university research review boards. The students were given no extra credit or compensation for participating. They were informed that their participation would explore their own learning processes. Students were invited to participate during the in-person teaching sessions of each master, and the response rate to the invitation was 96.32%.

### Instrument

To evaluate the conceptions of learning of the postgraduate students at the beginning of their master studies, a modified validated questionnaire previously developed by Purdie and Hattie [8] was adopted. The six general factors of conception originally identified by these authors with their respective items were used: (a) gaining information (INFO); (b) remembering, using, and understanding information (RUU); (c) a duty (DUTY); (d) personal change (PERS); (e) a process not bound by time or place (PROC) and (f) social competence (SOC). The original COLI was previously validated and showed reliability for the six factors (from 0.65 to 0.83 in the exploratory sample and from 0.50 to 0.86 in the validation sample) and internal consistency reliability index as determined by the Cronbach’s alpha (mean of 0.74) [8]. In addition, a 7th factor was evaluated by adding 10 novel items regarding the acquisition of professional competences (PROF) (Additional file 1: Table S1). This new factor was submitted to the validation analysis below described. We have used Spanish language -native for the students- instead of English. Participants were asked to rate their responses to a total of 42 items using a 7-point rating scale ranging from 1 (strongly disagree) to 7 (strongly agree), with the highest scores corresponding to responses that were more favorable and the lowest scores corresponding to more unfavorable responses. The questionnaire was filled during the first day session of each master. The students were first briefed on the purpose of the instrument and given instructions about how to complete the questionnaire.

### Statistical analysis

First, we carried out a reliability analysis of the whole questionnaire and an exploratory and confirmatory factor analysis to validate the new PROF factor of the questionnaire. Reliability and internal consistence of each factor in the questionnaire was evaluated by the alpha coefficient of Cronbach. The exploratory factor analysis using principal components extraction with the aim to validate the new added PROF factor was used. After the extraction, the number of factors with eigenvalues greater than 1.0 (Kaiser’s rule) that were explainable after varimax rotation were considered. Afterwards, a confirmatory factor analysis was performed in order to verify the one-dimensional structure of the obtained scale. Standardized residues were used to test the global adjust to the proposed model. Then, a bi-variate correlation matrix of the average scores of each factor was calculated and tested to determine the scale-level correlation among them.

Secondly, we analyzed the results obtained using the questionnaire to identify differences among the different study groups. Average values and standard deviations were calculated for each item, for each group of students and for each gender. Mean values were also calculated for each factor of items. Two-way ANOVA was used to compare the results obtained for each factor by gender and area (i.e., males vs. females and students corresponding to different master programs). For this analysis, the interaction between gender and area was firstly considered by performing the comparisons by gender for each area and by areas for each gender with Bonferroni’s penalty if the interaction was statistically significant. For factors whose interaction remain non-significant, an independent analysis was carried out for gender and area by performing pair-comparison. Neperian logarithm’s transformation (or square root transformation) of the variables was used to avoid mismatches in the ANOVA model (non-normality or heterogeneity of variances). *P* values below 0.05 were considered statistically significant for the two-tailed tests.

## Results

Here, we first analyzed the new PROF factor of the questionnaire to determine its validity. Exploratory factor analysis by principal components of the PROF factor found a solution of three components following the Kaiser’s rule with the first of these factors explaining 43.74% of the global variance. The other two components justified the remaining 25.27% of global variance. However, these two residual components were not considered as they referred to 2-item scales that were not solvable after rotation. Thus, a single component scale of 6 items was arranged with the items in Table 1 showing factor loadings. This component represent 54.50% of the 6-item scale variance. Verification of the one-dimensional structure of the scale was carried out by confirmatory factor analysis. Standardized residues ranged from − 0.79 to + 1.27 showing a robust fit between data and single-factor scale model. Thus, PROF factor was finally constituted by arranging items 1, 2, 3, 4, 5 and 8.

**Table 1:** Factor loading matrix of rotated components extracted by exploratory factor analysis of principal components applied in PROF factor

	Component		
	1	2	3
Item 7.1	**0.680**	0.371	
Item 7.2	**0.794**		
Item 7.3	**0.867**		
Item 7.4	**0.794**		
Item 7.5	**0.781**		
Item 7.6		0.344	0.777
Item 7.7		0.822	0.341
Item 7.8	**0.610**		
Item 7.9		0.827	
Item 7.10			0.808

Bold-type indicates items selected for the composition of PROF factor after exploratory factor anaysis

Then, we used a scale-level correlation analysis to compare the new PROF factor with the rest of factors included in original COLI by using a correlation matrix. Results of the correlation coefficients and *p*-values for the pairwise correlation between the PROF factor and each of the other 6 factors are shown in Table 2.

**Table 2:** Scale-level correlational analysis between PROF factor and each factor originally included in the COLI

	Pearson’s correlation	INFO	RUU	DUTY	PERS	PROC	SOC
PROF	ρ	0.566	0.541	0.369	0.373	0.284	0.429
	*p*-value	< 0.001	< 0.001	< 0.001	< 0.001	0.001	< 0.001

Results of alpha coefficient of Cronbach showed high reliability levels of each factor as shown in Table 3. Reliability ranged from α=0.6317 for DUTY factor to α=0.8734 for PROF factor. The alpha coefficient of Cronbach for the whole questionnaire was highly accurate and reliable (α=0.9216).

**Table 3:** Reliability and internal consistence of each factor in the questionnaire

	Alpha coefficient of Cronbach
INFO	0.7248
RUU	0.8412
DUTY	0.6317
PERS	0.8558
PROC	0.7054
SOC	0.8572
PROF	0.8734

Then, analysis of the average results obtained for each factor showed that the students rated with the highest values the items included in the PROC factor (average 6.23 ± 0.82), whereas the lowest values were found for the DUTY factor (5.13 ± 1.15). First, results of the two-way ANOVA analysis suggest that the gender of the students may influence conceptions of learning, mainly for the PERS and SOC factors, whereas the area influenced mainly the RUU factor. The interaction of gender and area did not show any significant influence on any of the factors (Table 4).

**Table 4:** Two-way ANOVA comparison of the scores in each factor

2-way ANOVA	INFO	RUU	DUTY	PERS	PROC	SOC	PROF
GENDER	0.273	0.662	0.129	0.027*	0.469	0.002*	0.533
AREA	0.054	0.005*	0.063	0.312	0.227	0.074	0.076
GENDER*AREA	0.605	0.817	0.812	0.421	0.521	0.088	0.578

Asterisk (*): *p* < 0.05

Table 5 shows the average results obtained for each master program and each factor. Statistical comparison of the average results obtained for each factor revealed specific differences between several master programs and between HS vs. NHS students (Tables 4 and 5 and Additional file 2: Table S2 and Additional file 3: Table S3). For INFO and DUTY, HS results were significantly higher than NHS and specifically, higher than SS results; for RUU, HS were not statistically different to NHS, although specific pairwise comparisons revealed that HS were significantly higher than SS. For SOC, HS results were higher than NHS, and pairwise comparison of HS vs. ES was statistically significant. No statistical differences were found among the master students for the PERS, PROC and PROF factors.

**Table 5:** Average ± standard deviation scores assigned to each factor of the questionnaire

	GENDER		AREA				
	Male	Female	HS	NHS			NHS
				ES	SS	AH	
INFO	5.17 ± 1.12	5.29 ± 1.09	5.74 ± 1.05^b,d^	5.34 ± 0.95	5.01 ± 1.02	4.99 ± 1.36	5.12 ± 1.13
RUU	5.36 ± 0.86	5.34 ± 0.94	5.43 ± 0.68^b^	5.67 ± 0.69	4.97 ± 0.86	5.33 ± 1.09	5.34 ± 0.93
DUTY	5.07 ± 1.13	5.24 ± 1.18	5.48 ± 1.03^b,d^	5.04 ± 1.00	4.77 ± 0.98	5.24 ± 1.43	5.02 ± 1.16
PERS	5.27 ± 0.97*	5.63 ± 0.96*	5.53 ± 0.86	5.20 ± 1.03	5.50 ± 0.94	5.54 ± 1.00	5.41 ± 0.99
PROC	6.19 ± 0.73	6.26 ± 0.91	6.10 ± 0.87	6.10 ± 0.73	6.28 ± 1.02	6.43 ± 0.63	6.27 ± 0.80
SOC	5.19 ± 1.29*	5.71 ± 1.00*	5.82 ± 0.81^a,d^	5.14 ± 1.33	5.35 ± 1.20	5.39 ± 1.22	5.29 ± 1.24
PROF	5.39 ± 0.91	5.44 ± 1.06	5.61 ± 0.79	5.60 ± 0.97	5.42 ± 0.86	5.16 ± 1.14	5.39 ± 1.01

* Males vs. females *p* < 0.05

^a^HS vs. ES *p* < 0.05

^b^HS vs. SS *p* < 0.05

^c^HS vs. AH *p* < 0.05

^d^HS vs. NHS *p* < 0.05

*HS* health sciences, *ES* experimental sciences, *AH* arts and humanities, *SS* social sciences, *NHS* non-health sciences

Bold means that statistical significant differences were found between male and female groups

Regarding gender differences, we found that the average results corresponding to female students were significantly higher than those corresponding to male students for the PERS and SOC factors. No differences were found between males and females for the rest of master programs for the average factors (Table 2 and Additional file 3: Table S3).

## Discussion

In the present work, we have analyzed the conceptions of learning of HS and NHS postgraduate students using the previously validated COLI questionnaire modified by the addition of a novel factor related to the acquisition of professional competences. As a result of this study, we have designed and validated a novel factor that could be available for other researchers with an interest in evaluating professional competences. In addition, we have demonstrated that both the gender and the area of knowledge of the students influence their conceptions of learning, although the effect of both variables was different.

Although the need to correlate medical education with learning theories has long been postulated [34], few substantial advances have been made in this area in undergraduate and postgraduate studies. Medical education research has mainly been focused on teaching methods, problem-based learning, and assessment of practicing physicians or continuing medical education [35, 36]. However, recent research has shown that students’ beliefs and conceptions of learning are relevant not only for their motivation but also for their learning strategies and achievement [11, 13]. According to Cliff [21] the studies carried out in this field have pointed out that identifying and classifying learners’ conceptions of learning may be a valid activity since distinct conceptions can be expression of different contexts and could drive to different learning outcomes.

Knowledge about postgraduate students’ conceptions of learning that could lead them to establish different motivations and learning strategies is necessary for a better understanding of the factors influencing their educational process. In addition, this knowledge could be useful to improve the didactic programs and the educational context to obtain a more appropriate academic achievement.

To investigate the conceptions of learning in postgraduate students and to determine the main factors that are influencing those conceptions, a comparative study was carried out with the conceptions of learning of master students of health sciences and other postgraduate programs in sciences, arts and humanities and social sciences. The selected programs have a very different nature, and students have a previous training that is also differentiated from secondary education [37]. This facilitates performing comparative studies that may be significant to evaluate the profiles of similarity that students share and other profiles that are more linked to the chosen postgraduate program according to their own conceptions of learning [20].

Purdie and Hattie had previously identified several factors involved in conception of learning and elaborated a new questionnaire called COLI (Conceptions of Learning Inventory) [8, 13]. Although it may have some limitations, this questionnaire is widely used [19] as it is considered to be an appropriate model for evaluating conception learning from different perspectives.

Although it is difficult to know which of the factors included in the questionnaire are related to a more superficial conception and which are with a deeper conception, the INFO factor is basically associated with the first one and the RUU and PERS factors, with the second one [8, 19].

As it is the case of previous studies using this questionnaire [19] the original inventory was modified to add a new factor linked to “learning as acquisition of professional competences” (PROF). We initially hypothesized that this factor could be relevant in the formative stage of postgraduate students rather than in previous educational levels, especially due to the immediate professional activity of students linked to postgraduate educational stages. This new version of the inventory kept high reliability as determined by the alpha coefficient of Cronbach obtained in our study. After designing this new PROF factor, we carried out a validation analysis. Because of this analysis, we found that 6 of the items originally included in PROF fulfilled the strict criteria that we set at the beginning (factor analysis). Therefore, in the present work, we have not only shed light on the differences between HS and NHS students, but we have also developed a novel factor concerning professional competences that showed very high reliability and accuracy and could be used in future works.

Regarding to the conception of learning as “gaining information” (INFO) we found that the average HS postgraduate results were significantly higher than NHS, and HS higher AH and SS students results. No statistical differences were observed between HS and ES students. This was especially evident for the specific item “learning helps me to become clever”. Although it has been shown that there are no differences between experimental scientists and social and humanities scientists in the information seeking [38], our study does show that there are differences in relation to the importance that students give to gaining information for their learning process. Gaining information was previously identified as an important issue for undergraduate health sciences students according to the perceptions of the students [35].

In relation to the concept of learning “as remembering, using, and understanding information (RUU)”, we did not find any global differences between HS and NHS, although our results showed that the average of items for HS and ES were significantly higher than SS. Scores assigned to all individual items were higher in HS and ES than in SS, being especially significant for items “when I have learned something, I know how to use it in other situations” and “learning is making sense out of new information and ways of doing things” in health sciences students. It is evident that these results, with no significant differences in RUU average scores assigned by HS and ES students are in agreement with those obtained in the previous factor INFO, with the highest average scores found for HS and ES in both factors. As suggested by [39], data and knowledge information should be focused on understanding, understood as a higher-order knowledge. Therefore, the close correlation found between these two factors of conceptions learning was expected. The fact that HS average scores were significantly higher than SS scores reveals a different pattern of conception learning in both types of students.

In “learning as a duty” (DUTY), the average of items for HS was also significantly higher than NHS, and HS higher than SS. Noteworthy, two of the three items included in DUTY (“learning is difficult but important” and “learning and studying must be done whether I like it or not”) showed significant differences between HS and SS students and between HS and ES. Surprisingly, no significant differences were found between HS and AH students. These results may be related to the high level of motivation and emotional intelligence generally attributed to health professionals. Emotional intelligence influences their ability to provide safe and compassionate health care. This ability is present prior to the beginning of their undergraduate training and is presumably increased during their postgraduate studies. If emotional intelligence is a trait, a learned ability or a combination of the two is discussed but is presumably an important component in the DUTY factor of conception of learning [40].

For the factor “Learning as the development of social competence” (SOC), the average of items in HS students was again higher than NHS students, and HS was higher as compared to ES students. This result is especially important because there are no significant differences between the average of HS and AH and SS. While the social approach is clearly present in the conception of learning of these students, a less social awareness there exists in the conception of learning of ES students. This suggests that ES students may conceive their learning as addressed to the search of pure scientific knowledge, whereas the social application of this knowledge could be secondly to them.

Our results did not show any statistical differences between the average scores obtained for each group of student for the PERS, PROC and PROF factors. This means that the incidence of these factors in conception of learning is similar for all students regardless of the program they belong to. In addition, our results show that the average of items included in the PROF factor were rated with the lowest values, which may be in conflict with our initial hypothesis. However, the highest average values corresponded to the PROC factor. This means that students enrolled in health sciences, clearly distinguish between a postgraduate master degree program, where learning is conceived as a process not bound by time or place -PROC factor-, and a postgraduate residency program, where learning is expected to take place at, through or from work in a work-specific place [41].

Some slight differences have been previously established in male and female students in relation to medical education, especially in motivation [31, 32]. However, we did not find any significant differences for the average values of factors related to conception of learning between male and female students, except for PERS and SOC in AH students, although some specific items showed differences for AH and SS students. These results may imply that conception of learning is not influenced by gender in HS students at the postgraduate level. If feminizing of medicine can be extended to medical education as suggested by Bleakley [42], and if it could influence in some way the postgraduate process of learning, is something that should be investigated in the future.

These finding are potentially useful to foster the learning process of HS students, because if they are metacognitively aware of their own conception or learning, they will be much better equipped to self-regulate their learning behavior in a postgraduate master program in health sciences. Furthermore, these results could help instructors as well as education policy makers to use this information to foster the main factors that influence the learning process of these students, not only at the cognitive and skill acquisition levels, but also to enhance the didactic methods to be used for this type of programs.

The present study has some strengths and limitations. One of the strengths is the identification of specific conceptions of learning of postgraduate students corresponding to all major areas of knowledge that allowed us to distinguish the specific features of HS students as compared to NHS. Another strength is the use of evaluation tools with acceptable reliability and accuracy, including a novel factor developed and validated by the authors that could be used in forthcoming research. Limitations are mainly related to the sample size, since the number of students enrolled in postgraduate master programs is always low. Future studies should be carried out with higher samples.

## Conclusions

In summary, our results show that the overall conceptions of learning differ among students of HS and NHS postgraduate master programs. The present study demonstrates that a set of factors may influence conception of learning of HS postgraduate students. Among these factors, learning as gaining information, remembering, using, and understanding information, awareness of duty and social commitment could be the most relevant. For these students, learning as a personal change, a process not bound by time or place or even as acquisition of professional competences, are less relevant. According to our results, this profile is not affected by gender differences.

## Additional files

Additional file 1: **Table S1.** Factor number 7 related to “Learning as acquisition of professional competences” (PROF) added to the Conceptions of Learning Inventory (COLI) originally developed by Purdie and Hattie. (PDF 174 kb)

Additional file 2: **Table S2.** Average ± standard deviation scores assigned to each item and to each factor of the questionnaire used in the present work and *p* values of the statistical comparisons between two specific master programs. *p* values < 0.05 are highlighted with asterisks (*). (PDF 348 kb)

Additional file 3: **Table S3.** Average ± standard deviation scores assigned to each item and to each factor for male and female students and p values of the statistical comparisons between genders for the students of each program using ANOVA (“*P* value” columns). Statistically significant p values are highlighted with asterisks (*). (PDF 375 kb)

## Abbreviations

AH: Arts and humanities
COLI: Conceptions of learning inventory
DUTY: Duty
ES: Experimental sciences
HS: Health sciences
INFO: Gaining information
PERS: Personal change
PROC: A process not bound by time or place
PROF: Acquisition of professional competences
RUU: Remembering, using, and understanding information
SOC: Social competence
SS: Social sciences

## References

[1] Klatter EB, Lodewijks HGLC, Aarnoutse CAJ (2001). Learning conceptions of young students in the final year of primary education. Learn Instr.

[2] Säljö R. Learning in the Learner’s perspective: some common-sense conceptions. Göteborg: Institute of Education: University of Göteborg; 1979.

[3] Boulton-Lewis GM, Marton F, Lewis DC, Wilss LA (2004). A longitudinal study of learning for a group of indigenous Australian university students: dissonant conceptions and strategies. High Educ.

[4] Entwistle N, Vosniadou S, Baltas S (2007). Conceptions of learning and teaching: relationships with study strategies and understanding. Philosophical, historical and psychological approaches to conceptual change.

[5] Entwistle N, Peterson E, Spielberg C (2004). Learning styles and approaches to studying. Encyclopedia of applied psychology.

[6] Hattie J, Yates GCR. Visible learning and the science of how we learn: Taylor & Francis. Abingdom: Routledge; 2013.

[7] Marton F, Dall'Alba G, Beaty E (1993). Conceptions of learning. Int J Educ Res.

[8] Purdie N, Hattie J (2002). Assessing students’ conceptions of learning. Aust J Educ Dev Psychol.

[9] Shuell TJ (1986). Cognitive conceptions of learning. Rev Educ Res.

[10] Vanrossum EJ, Deijkers R, Hamer R (1985). Students learning conceptions and their interpretation of significant educational concepts. High Educ.

[11] Alamdarloo GH, Moradi S, Dehshiri GR (2013). The relationship between students’ conceptions of learning and their academic achievement. Psychology.

[12] Hong YY, Salili F (2000). Challenges ahead for research on Chinese students’ learning motivation in the new millennium. JPCS.

[13] Purdie N, Hattie J, Douglas G (1996). Student conceptions of learning and their use of self-regulated learning strategies: a cross-cultural comparison. J Educ Psychol.

[14] Bernardo AB (2003). Approaches to learning and academic achievement of Filipino students. J Genet Psychol.

[15] McLean M (2001). Can we relate conceptions of learning to student academic achievement?. Teach High Educ.

[16] Jacobs JC, van Luijk SJ, Galindo-Garre F, Muijtjens AM, van der Vleuten CP, Croiset G (2014). Five teacher profiles in student-centred curricula based on their conceptions of learning and teaching. BMC Med Educ.

[17] Jacobs JC, van Luijk SJ, van der Vleuten CP, Kusurkar RA, Croiset G, Scheele F (2016). Teachers’ conceptions of learning and teaching in student-centred medical curricula: the impact of context and personal characteristics. BMC Med Educ.

[18] Jacobs JCG, Van Luijk SJ, Van Berkel H, Van der Vleuten CPM, Croiset G, Scheele F (2012). Development of an instrument (the COLT) to measure conceptions on learning and teaching of teachers, in student-centred medical education. Med Teach.

[19] Peterson ER, Brown GTL, Irving SE (2010). Secondary school students’ conceptions of learning and their relationship to achievement. Learn Individ Differ.

[20] Boulton-Lewis GM (2004). Conceptions of teaching and learning at school and university : similarities, differences, relationships and contextual factors. Eur J School Psychol.

[21] Cliff AF (1998). Teacher-learners’ conceptions of learning: evidence of a “communalist” conception amongst postgraduate learners?. High Educ.

[22] Richardson JTE (1994). Mature students in higher-education .1. A literature survey on approaches to studying. Stud High Educ.

[23] Richardson JTE (2011). Approaches to studying, conceptions of learning and learning styles in higher education. Learn Individ Differ.

[24] Haggis T (2002). Exploring the ‘black box’ of process: a comparison of theoretical notions of the ‘adult learner’ with accounts of postgraduate learning experience. Stud High Educ.

[25] Murad MH, Varkey P (2008). Self-directed learning in health professions education. Ann Acad Med Singap.

[26] Sawatsky AP, Ratelle JT, Bonnes SL, Egginton JS, Beckman TJ (2017). A model of self-directed learning in internal medicine residency: a qualitative study using grounded theory. BMC Med Educ.

[27] ten Cate O (2005). Entrustability of professional activities and competency-based training. Med Educ.

[28] ten Cate O, Scheele F (2007). Competency-based postgraduate training: can we bridge the gap between theory and clinical practice?. Acad Med.

[29] Sola M, Sánchez-Martínez M, Ruyffelaert A, Campos F, Carriel V, Rodríguez I (2015). Identification of learning conceptions in postgraduate students of health sciences and social sciences. Actual Med.

[30] Meyer JHF, Boulton-Lewis G (1997). Variation in students’ conceptions of learning: an exploration of cultural and discipline effects. Research and development in. High Educ.

[31] Campos-Sanchez A, Lopez-Nunez JA, Carriel V, Martin-Piedra MA, Sola T, Alaminos M (2014). Motivational component profiles in university students learning histology: a comparative study between genders and different health science curricula. BMC Med Educ.

[32] Kusurkar R, Kruitwagen C, ten Cate O, Croiset G (2010). Effects of age, gender and educational background on strength of motivation for medical school. Adv Health Sci Educ Theory Pract.

[33] Chiou GL, Liang JC, Tsai CC (2012). Undergraduate Students’ conceptions of and approaches to learning in biology: a study of their structural models and gender differences. Int J Sci Educ.

[34] Bleakley A (2006). Broadening conceptions of learning in medical education: the message from teamworking. Med Educ.

[35] Campos-Sanchez A, Martin-Piedra MA, Carriel V, Gonzalez-Andrades M, Garzon I, Sanchez-Quevedo MC (2012). Reception learning and self-discovery learning in histology: students’ perceptions and their implications for assessing the effectiveness of different learning modalities. Anat Sci Educ.

[36] Norman G (2002). Research in medical education: three decades of progress. BMJ.

[37] Dart BC, Burnett PC, Purdie N, Boulton-Lewis G, Campbell J, Smith D (2000). Students’ conceptions of learning, the classroom environment, and approaches to learning. J Educ Res.

[38] Ellis D, Cox D, Hall K (1993). A comparison of the information-seeking patterns of researchers in the physical and social-sciences. J Doc.

[39] Bawden D, Robinson L (2016). Information and the gaining of understanding. J Inf Sci.

[40] Cherry MG, Fletcher I, O'Sullivan H, Dornan T (2014). Emotional intelligence in medical education: a critical review. Med Educ.

[41] Swanwick T (2005). Informal learning in postgraduate medical education: from cognitivism to ‘culturism’. Med Educ.

[42] Bleakley A (2013). Gender matters in medical education. Med Educ.

