# Supplementary material for: Conceptions of learning factors in postgraduate health sciences master students: a comparative study with non-health science students and between genders
Source: BMC Med Educ. 2018 Jun 7;18:128. doi: 10.1186/s12909-018-1227-x (PMC5992711; doi:10.1186/s12909-018-1227-x)
Supplement: Supplementary file 1 — Table S1. Factor number 7 related to “Learning as acquisition of professional competences” (PROF) added to the Conceptions of Learning Inventory (COLI) originally developed by Purdie and Hattie. (PDF 174 kb) [file 12909_2018_1227_MOESM1_ESM.pdf]

Additional Table S1

Factor number 7 related to “Learning as acquisition of professional competences” (PROF) added to the Conceptions of Learning Inventory (COLI) originally developed by Purdie and Hattie.

**7. Learning as acquisition of professional competences (PROF)**
7.1. Learning a master is acquiring concept competences
7.2. Learning a master is acquiring attitudinal competences
7.3. Learning a master is acquiring skill competences
7.4. Learning a master is learning skills and abilities
7.5. Learning a master is learning specialized competences for a labor activity
7.6. Learning a master is acquiring only basic competences for further learning
7.7. Learning a master is acquiring experience by repeating previously carried out procedures
7.8. Learning a master is acquiring experience in thinking how to solve new problems.
7.9. Learning a master is to follow the instructions of an instructor
7.10. Learning a master is a self-regulated self-learning process
