# Supplementary material for: Conceptions of learning factors in postgraduate health sciences master students: a comparative study with non-health science students and between genders
Source: BMC Med Educ. 2018 Jun 7;18:128. doi: 10.1186/s12909-018-1227-x (PMC5992711; doi:10.1186/s12909-018-1227-x)
Supplement: Supplementary file 2 — Table S2. Average ± standard deviation scores assigned to each item and to each factor of the questionnaire used in the present work and p values of the statistical comparisons between two specific master programs. p values < 0.05 are highlighted with asterisks (*). (PDF 348 kb) [file 12909_2018_1227_MOESM2_ESM.pdf]

Additional Table S2. Average ± standard deviation scores assigned to each item and to each factor of the questionnaire used in the present work and p values of the statistical comparisons between two specific master programs. p values <0.05 are highlighted with asterisks (*).

		AH	ES	HS	SS	AH vs. ES	AH vs. HS	AH vs. SS	ES vs. HS	ES vs. SS	HS vs. SS
INFO	ITEM 1.1	4.76±1.84	5.17±1.50	6.00±3.83	4.77±1.61	0.3183	0.0966	0.9964	0.2402	0.2995	0.1075
	ITEM 1.2	4.44±2.19	4.63±1.75	4.84±1.63	4.40±1.65	0.6955	0.4024	0.9333	0.6052	0.5921	0.2913
	ITEM 1.3	5.62±1.65	5.51±1.25	5.78±1.24	5.50±1.31	0.7696	0.6519	0.7553	0.3826	0.9642	0.3875
	ITEM 1.4	4.24±2.09	5.31±1.35	5.84±1.02	4.63±1.61	0.0128*	0.0002*	0.4012	0.0761	0.0677	0.0007*
	ITEM 1.5	5.88±1.15	6.09±0.92	6.25±0.76	5.73±1.11	0.4190	0.1328	0.6010	0.4312	0.1669	0.0360*
	**MEAN 1**	**4.99±1.36**	**5.34±0.95**	**5.74±1.05**	**5.01±1.02**	**0.2127**	**0.0144***	**0.9518**	**0.1055**	**0.1742**	**0.0068***
RUU	ITEM 2.1	4.71±1.78	5.63±1.42	5.34±1.26	4.97±1.56	0.0200*	0.1002	0.5389	0.3894	0.0782	0.2987
	ITEM 2.2	4.29±1.80	4.91±1.72	4.28±1.61	3.93±1.64	0.1483	0.9757	0.4075	0.1260	0.0224*	0.4027
	ITEM 2.3	4.71±1.40	5.17±1.15	4.56±1.29	3.90±1.49	0.1361	0.6681	0.0298*	0.0455*	0.0003*	0.0663
	ITEM 2.4	5.06±1.67	5.49±0.89	4.94±1.37	4.67±1.45	0.1873	0.7485	0.3221	0.0538	0.0069*	0.4513
	ITEM 2.5	5.79±1.25	5.86±1.03	5.75±0.84	5.17±1.37	0.8199	0.8678	0.0597	0.6452	0.0238*	0.0460*
	ITEM 2.6	6.09±1.11	6.09±0.92	6.19±0.69	5.73±1.17	0.9918	0.6669	0.2187	0.6132	0.1796	0.0662
	ITEM 2.7	6.00±1.23	5.83±1.15	6.13±0.66	5.53±1.04	0.5519	0.6121	0.1092	0.2059	0.2855	0.0093*
	ITEM 2.8	5.91±1.29	6.14±1.09	6.06±1.16	5.73±1.34	0.4232	0.6201	0.5889	0.7710	0.1784	0.3042
	ITEM 2.9	5.44±1.69	5.91±0.92	5.59±1.01	5.13±1.28	0.1519	0.6605	0.4196	0.1789	0.0058*	0.1200
	**MEAN 2**	**5.33±1.09**	**5.67±0.69**	**5.43±0.68**	**4.97±0.86**	**0.1284**	**0.6781**	**0.1519**	**0.1509**	**0.0006***	**0.0243***
DUTY	ITEM 3.1	5.41±1.76	4.86±1.52	5.59±1.46	4.73±1.34	0.1652	0.6499	0.0908	0.0472*	0.7303	0.0186*
	ITEM 3.2	5.94±1.39	6.23±0.97	6.03±1.00	5.63±0.96	0.3224	0.7648	0.3139	0.4160	0.0163*	0.1163
	ITEM 3.3	4.35±2.23	4.03±1.65	4.81±1.42	3.93±1.95	0.4937	0.3252	0.4283	0.0424*	0.8318	0.0459*
	**MEAN 3**	**5.24±1.43**	**5.04±1.00**	**5.48±1.03**	**4.77±0.98**	**0.5084**	**0.4335**	**0.1372**	**0.0800**	**0.2739**	**0.0072***
PERS	ITEM 4.1	6.68±0.73	6.26±1.01	6.39±0.84	6.37±0.81	0.0525	0.1426	0.1116	0.5753	0.6350	0.9235
	ITEM 4.2	6.06±1.10	5.83±1.18	6.19±1.03	5.97±1.07	0.4040	0.6258	0.7354	0.1901	0.6239	0.4101
	ITEM 4.3	5.71±1.31	5.51±1.25	5.41±1.10	5.90±1.30	0.5364	0.3212	0.5551	0.7093	0.2264	0.1107
	ITEM 4.4	6.09±1.14	5.71±1.10	5.69±1.06	6.07±1.08	0.1697	0.1444	0.9385	0.9196	0.1990	0.1685
	ITEM 4.5	4.47±2.08	3.97±1.72	4.75±1.41	4.60±1.71	0.2807	0.5279	0.7884	0.0485*	0.1466	0.7076
	ITEM 4.6	5.74±1.46	5.23±1.44	5.81±0.93	5.53±1.36	0.1513	0.8003	0.5708	0.0550	0.3852	0.3463
	ITEM 4.7	4.56±2.08	4.63±1.46	5.13±1.24	4.83±1.53	0.8719	0.1867	0.5542	0.1395	0.5833	0.4117
	ITEM 4.8	5.06±1.70	4.49±1.88	4.91±1.42	4.73±1.62	0.1901	0.6952	0.4379	0.3099	0.5752	0.6560
	**MEAN 4**	**5.54±1.00**	**5.20±1.03**	**5.53±0.86**	**5.50±0.94**	**0.1677**	**0.9594**	**0.8570**	**0.1616**	**0.2324**	**0.8878**
PROC	ITEM 5.1	6.82±0.46	6.60±0.65	6.47±0.88	6.47±1.11	0.1047	0.0422*	0.0899	0.4874	0.5491	0.9935
	ITEM 5.2	6.38±1.02	6.14±0.97	6.09±1.06	6.33±1.12	0.3210	0.2624	0.8552	0.8439	0.4670	0.3909
	ITEM 5.3	6.09±1.16	5.57±1.31	5.75±1.08	6.03±1.33	0.0885	0.2259	0.8605	0.5471	0.1641	0.3581
	**MEAN 5**	**6.43±0.63**	**6.10±0.73**	**6.10±0.87**	**6.28±1.02**	**0.0501**	**0.0822**	**0.4656**	**0.9976**	**0.4295**	**0.4720**
SOC	ITEM 6.1	5.38±1.41	4.91±1.58	5.59±1.13	4.97±1.38	0.1995	0.5067	0.2394	0.0488*	0.8880	0.0541
	ITEM 6.2	6.15±1.12	5.86±1.31	6.19±0.82	6.00±1.39	0.3244	0.8834	0.6342	0.2254	0.6713	0.5170
	ITEM 6.3	5.18±1.71	5.11±1.55	5.91±0.86	5.40±1.43	0.8748	0.0340*	0.5759	0.0129*	0.4452	0.0934
	ITEM 6.4	4.88±1.70	4.69±1.71	5.59±1.01	5.03±1.65	0.6338	0.0446*	0.7206	0.0112*	0.4097	0.1097
	**MEAN 6**	**5.39±1.22**	**5.14±1.33**	**5.82±0.81**	**5.35±1.20**	**0.4235**	**0.0975**	**0.8959**	**0.0152***	**0.5137**	**0.0733**
PROF	ITEM 7.1	5.00±1.33	4.89±1.39	5.44±1.19	4.87±1.36	0.7278	0.1640	0.6927	0.0868	0.9558	0.0829
	ITEM 7.2	4.82±1.49	5.29±1.20	5.53±1.14	5.27±1.20	0.1597	0.0343*	0.1984	0.3944	0.9494	0.3762
	ITEM 7.3	5.38±1.37	5.89±1.05	5.78±0.94	5.43±1.01	0.0910	0.1756	0.8674	0.6707	0.0825	0.1647
	ITEM 7.4	5.35±1.47	6.11±1.08	5.72±0.92	5.60±1.19	0.0168*	0.2350	0.4677	0.1134	0.0726	0.6615
	ITEM 7.5	5.29±1.40	5.60±1.33	5.69±0.78	5.87±0.86	0.3566	0.1679	0.0576	0.7470	0.3507	0.3933
	ITEM 7.6	4.68±1.98	4.91±1.50	4.75±1.24	4.33±1.97	0.5753	0.8583	0.4908	0.6294	0.1829	0.3204
	ITEM 7.7	3.74±1.64	4.23±1.50	4.38±1.26	3.80±1.58	0.1959	0.0818	0.8733	0.6682	0.2669	0.1183
	ITEM 7.8	5.09±1.40	5.80±1.30	5.53±0.95	5.50±1.48	0.0322*	0.1400	0.2574	0.3418	0.3878	0.9210
	ITEM 7.9	3.65±1.43	3.37±1.57	3.88±1.31	3.03±1.54	0.4498	0.5038	0.1040	0.1619	0.3867	0.0239*
	ITEM 7.10	4.85±1.64	5.26±1.15	5.25±1.16	4.57±1.22	0.2376	0.2627	0.4358	0.9799	0.0221*	0.0278*
	**MEAN 7**	**4.79±1.00**	**5.13±0.84**	**5.19±0.74**	**4.83±0.81**	**0.1216**	**0.0655**	**0.8577**	**0.7605**	**0.1396**	**0.0667**
