# Supplementary material for: Conceptions of learning factors in postgraduate health sciences master students: a comparative study with non-health science students and between genders
Source: BMC Med Educ. 2018 Jun 7;18:128. doi: 10.1186/s12909-018-1227-x (PMC5992711; doi:10.1186/s12909-018-1227-x)
Supplement: Supplementary file 3 — Table S3. Average ± standard deviation scores assigned to each item and to each factor for male and female students and p values of the statistical comparisons between genders for the students of each program using ANOVA (“P value” columns). Statistically significant p values are highlighted with asterisks (*). (PDF 375 kb) [file 12909_2018_1227_MOESM3_ESM.pdf]

Additional Table S3. Average ± standard deviation scores assigned to each item and to each factor for male and female students and p values of the statistical comparisons between genders for the students of each program using ANOVA (“P value” columns). Statistically significant p values are highlighted with asterisks (*).

		AH			ES			HS			SS		
		MALES	FEMALES	P value	MALES	FEMALES	P value	MALES	FEMALES	P value	MALES	FEMALES	P value
INFO	ITEM 1.1	4.62±1.86	5.00±1.87	0.5662	5.06±1.47	5.29±1.57	0.6461	6.10±4.71	5.82±0.98	0.8494	5.17±1.83	4.13±1.64	0.2221
	ITEM 1.2	3.95±2.09	5.23±2.20	0.0988	4.33±1.64	4.94±1.85	0.3116	5.00±1.55	4.55±1.81	0.4624	4.33±2.16	4.07±1.75	0.7707
	ITEM 1.3	5.48±1.75	5.85±1.52	0.5339	5.83±0.99	5.18±1.42	0.1204	5.71±1.31	5.91±1.14	0.6794	4.17±1.60	5.87±1.06	0.0098*
	ITEM 1.4	4.00±2.02	4.62±2.22	0.4124	5.17±1.38	5.47±1.33	0.5123	5.90±1.04	5.73±1.01	0.6476	3.83±1.17	4.73±1.83	0.2819
	ITEM 1.5	5.67±1.11	6.23±1.17	0.1674	6.22±0.81	5.94±1.03	0.3740	6.38±0.74	6.00±0.77	0.1835	5.67±1.03	5.73±1.03	0.8951
	MEAN 1	4.74±1.38	5.38±1.27	0.1852	5.32±0.82	5.36±1.10	0.8973	5.82±1.14	5.60±0.87	0.5827	4.63±1.17	4.91±1.06	0.6104
RUU	ITEM 2.1	4.62±1.91	4.85±1.63	0.7243	5.56±1.58	5.71±1.26	0.7587	5.38±1.32	5.27±1.19	0.8218	4.67±1.21	4.80±1.86	0.8737
	ITEM 2.2	4.00±1.84	4.77±1.69	0.2317	4.61±1.88	5.24±1.52	0.2903	4.29±1.85	4.27±1.10	0.9831	3.67±0.82	3.53±1.81	0.8654
	ITEM 2.3	4.52±1.36	5.00±1.47	0.3443	5.22±1.00	5.12±1.32	0.7925	4.62±1.43	4.45±1.04	0.7387	2.83±1.33	3.73±1.39	0.1904
	ITEM 2.4	5.10±1.73	5.00±1.63	0.8744	5.39±0.85	5.59±0.94	0.5144	4.95±1.40	4.91±1.38	0.9338	4.00±1.79	4.47±1.36	0.5223
	ITEM 2.5	5.86±1.20	5.69±1.38	0.7147	6.00±0.59	5.71±1.36	0.4080	5.67±0.91	5.91±0.70	0.4485	5.67±1.03	4.67±1.59	0.1732
	ITEM 2.6	6.14±1.11	6.00±1.15	0.7216	6.00±1.08	6.18±0.73	0.5781	6.19±0.75	6.18±0.60	0.9739	6.33±0.82	5.27±1.28	0.0758*
	ITEM 2.7	5.86±1.28	6.23±1.17	0.3980	5.83±1.38	5.82±0.88	0.9803	6.14±0.73	6.09±0.54	0.8365	5.33±0.82	5.60±1.12	0.6050
	ITEM 2.8	5.67±1.35	6.31±1.11	0.1616	6.11±1.13	6.18±1.07	0.8622	6.14±1.35	5.91±0.70	0.5972	5.33±1.03	5.67±1.59	0.6425
	ITEM 2.9	5.14±1.90	5.92±1.19	0.1955	5.94±0.87	5.88±0.99	0.8452	5.71±0.96	5.36±1.12	0.3601	5.00±1.55	4.87±1.25	0.8381
	MEAN 2	5.21±1.09	5.53±1.11	0.4158	5.63±0.71	5.71±0.69	0.7276	5.46±0.73	5.37±0.59	0.7529	4.76±0.43	4.73±1.01	0.9529
DUTY	ITEM 3.1	5.00±1.76	6.08±1.61	0.0828	4.61±1.85	5.12±1.05	0.3310	5.43±1.33	5.91±1.70	0.3840	4.00±1.67	5.07±0.88	0.0690
	ITEM 3.2	6.05±1.12	5.77±1.79	0.5787	5.94±1.21	6.53±0.51	0.0749	6.10±0.83	5.91±1.30	0.6247	5.33±0.82	5.47±1.13	0.7960
	ITEM 3.3	4.38±2.40	4.31±2.02	0.9274	3.72±1.78	4.35±1.50	0.2656	4.86±1.28	4.73±1.74	0.8110	3.67±1.63	3.93±1.98	0.7740
	MEAN 3	5.14±1.33	5.38±1.63	0.6402	4.76±1.15	5.33±0.72	0.0884	5.46±0.81	5.52±1.42	0.8894	4.33±0.82	4.82±0.96	0.2866
PERS	ITEM 4.1	6.62±0.80	6.77±0.60	0.5663	6.17±1.25	6.35±0.70	0.5931	6.30±0.92	6.55±0.69	0.4477	6.33±1.03	6.60±0.51	0.4307
	ITEM 4.2	5.86±1.06	6.38±1.12	0.1778	5.72±1.32	5.94±1.03	0.5894	6.24±0.83	6.09±1.38	0.7076	5.67±1.21	6.07±1.22	0.5054
	ITEM 4.3	5.43±1.29	6.15±1.28	0.1196	5.28±1.53	5.76±0.83	0.2536	5.29±1.10	5.64±1.12	0.4019	5.50±1.52	5.93±1.44	0.5459
	ITEM 4.4	5.76±1.18	6.62±0.87	0.0313*	5.78±1.06	5.65±1.17	0.7310	5.62±1.07	5.82±1.08	0.6219	5.83±0.98	6.13±1.36	0.6300
	ITEM 4.5	4.14±2.15	5.00±1.91	0.2484	3.44±1.79	4.53±1.50	0.0616	4.81±1.47	4.64±1.36	0.7480	3.67±1.03	4.67±1.76	0.2113
	ITEM 4.6	5.43±1.57	6.23±1.17	0.1218	5.00±1.64	5.47±1.18	0.3403	5.76±0.94	5.91±0.94	0.6782	5.17±1.83	5.93±0.96	0.2201
	ITEM 4.7	3.95±2.16	5.54±1.56	0.0281*	4.67±1.57	4.59±1.37	0.8763	5.29±1.10	4.82±1.47	0.3181	4.83±1.60	4.87±1.77	0.9685
	ITEM 4.8	4.86±1.71	5.38±1.71	0.3888	4.11±2.11	4.88±1.58	0.2316	5.10±1.41	4.55±1.44	0.3067	3.50±1.38	5.13±1.55	0.0371*
	MEAN 4	5.26±0.94	6.01±0.95	0.0306*	5.02±1.12	5.40±0.90	0.2850	5.55±0.84	5.50±0.93	0.8801	5.06±1.03	5.67±1.03	0.2403
PROC	ITEM 5.1	6.86±0.36	6.77±0.60	0.5948	6.61±0.61	6.59±0.71	0.9190	6.48±0.87	6.45±0.93	0.9486	6.33±1.21	6.53±1.30	0.7496
	ITEM 5.2	6.19±1.21	6.69±0.48	0.1647	6.33±0.84	5.94±1.09	0.2396	6.10±1.09	6.09±1.04	0.9914	6.17±0.75	6.53±0.92	0.3968
	ITEM 5.3	5.95±1.32	6.31±0.85	0.3955	5.50±1.34	5.65±1.32	0.7458	5.90±0.94	5.45±1.29	0.2685	5.33±1.37	6.13±1.46	0.2624
	MEAN 5	6.33±0.67	6.59±0.55	0.2532	6.15±0.62	6.06±0.84	0.7220	6.16±0.83	6.00±0.97	0.6299	5.94±0.95	6.40±1.14	0.4001
SOC	ITEM 6.1	5.00±1.55	6.00±0.91	0.0433*	4.56±1.79	5.29±1.26	0.1700	5.57±1.25	5.64±0.92	0.8805	3.67±1.51	5.47±0.99	0.0043*
	ITEM 6.2	5.90±1.21	6.54±0.88	0.1112	5.72±1.41	6.00±1.22	0.5385	6.33±0.73	5.91±0.94	0.1685	5.00±2.19	6.40±0.83	0.0421
	ITEM 6.3	4.81±1.81	5.77±1.42	0.1138	4.78±1.66	5.47±1.37	0.1900	5.95±0.74	5.82±1.08	0.6807	4.67±1.63	5.80±1.15	0.0853
	ITEM 6.4	4.43±1.78	5.62±1.33	0.0463*	4.33±1.78	5.06±1.60	0.2148	5.67±0.97	5.45±1.13	0.5816	4.00±2.19	5.47±1.64	0.1084
	MEAN 6	5.02±1.24	5.98±0.95	0.0233*	4.85±1.40	5.46±1.20	0.1784	5.88±0.79	5.70±0.89	0.5676	4.33±1.69	5.78±0.88	0.0171
PROF	ITEM 7.1	4.95±1.16	5.08±1.61	0.7947	4.72±1.49	5.06±1.30	0.4817	5.38±1.32	5.55±0.93	0.7167	4.67±1.75	4.73±1.28	0.9235
	ITEM 7.2	4.76±1.41	4.92±1.66	0.7639	5.28±1.23	5.29±1.21	0.9687	5.43±1.25	5.73±0.90	0.4887	4.50±1.38	5.13±1.19	0.3038
	ITEM 7.3	5.57±1.21	5.08±1.61	0.3141	5.78±1.22	6.00±0.87	0.5398	5.76±0.94	5.82±0.98	0.8755	5.33±1.21	5.33±1.11	1.0000
	ITEM 7.4	5.81±0.93	4.62±1.89	0.0193	5.94±1.30	6.29±0.77	0.3453	5.67±0.91	5.82±0.98	0.6669	5.00±1.55	5.53±1.19	0.4036
	ITEM 7.5	5.33±1.20	5.23±1.74	0.8397	5.44±1.50	5.76±1.15	0.4856	5.62±0.80	5.82±0.75	0.5019	5.67±1.03	5.87±0.83	0.6472
	ITEM 7.6	4.95±2.09	4.23±1.79	0.3091	4.83±1.38	5.00±1.66	0.7482	4.57±1.33	5.09±1.04	0.2689	3.83±2.14	4.27±2.34	0.6998
	ITEM 7.7	3.48±1.60	4.15±1.68	0.2472	4.06±1.51	4.41±1.50	0.4898	4.24±1.48	4.64±0.67	0.4060	3.67±1.51	3.60±1.59	0.9309
	ITEM 7.8	5.19±1.08	4.92±1.85	0.5963	5.67±1.64	5.94±0.83	0.5409	5.62±1.02	5.36±0.81	0.4791	4.33±1.21	5.40±1.59	0.1582
	ITEM 7.9	3.62±1.28	3.69±1.70	0.8874	3.28±1.53	3.47±1.66	0.7229	3.71±1.49	4.18±0.87	0.3473	2.67±1.37	2.73±1.62	0.9305
	ITEM 7.10	4.86±1.62	4.85±1.72	0.9852	5.22±0.94	5.29±1.36	0.8561	5.14±1.31	5.45±0.82	0.4809	4.00±1.79	4.80±1.21	0.2462
	MEAN 7	4.85±0.75	4.68±1.34	0.6274	5.02±0.82	5.25±0.88	0.4256	5.11±0.82	5.35±0.55	0.4097	4.37±1.04	4.74±0.76	0.3702
